# Former Mucinous Bronchioloalveolar Carcinoma Revisited

**DOI:** 10.1155/2013/284323

**Published:** 2013-11-28

**Authors:** Mohammed H. AlShati, Mohammed M. Yaktien, Kenneth C. Katchy

**Affiliations:** ^1^Division of Respirology and Sleep Medicine, Department of Internal Medicine, Al-Adan Hospital, P.O. Box 46969, Ahmadi 64020, Kuwait; ^2^Department of Radiology, Al-Adan Hospital, P.O. Box 46969, Ahmadi 64020, Kuwait; ^3^Department of Pathology, Al-Sabah Hospital, P.O. Box 4078, Safat 13041, Kuwait

## Abstract

This is a brief case report of invasive multicentric mucinous adenocarcinoma presented at a rather young age with bronchorrhea and persistent consolidation that ended up with the patient demise; nevertheless, we demonstrate relevant radiological and pathological features with emphasis on the new classification of bronchioloalveolar carcinoma, a term that should no longer be in use.

## 1. Case

**Figure 1:**
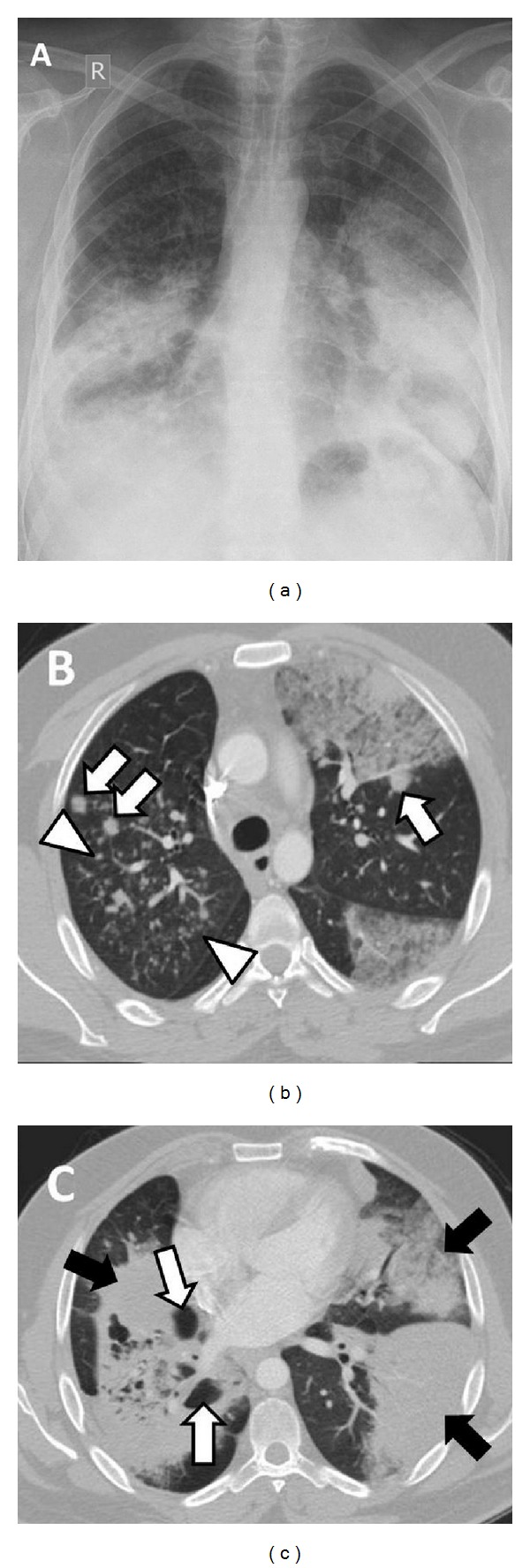
(a) Plain posteroanterior chest radiograph showing diffuse bilateral mid and lower lung zone consolidation. (b) Axial CT scan (lung window, slice thickness: 2 mm) showing scattered nodules (arrows) combined with multifocal consolidation, ground glass opacities, and septal wall thickening. Centrilobular nodules are also evident in the right lung (between arrow heads) and thought to represent aerogenous spread of tumor cells. (c) Diffuse bilateral multilobar consolidation (black arrows) with the right lower lobe showing a mixture of consolidation, cystic airspaces, and pseudocavitations (white arrows).

A 35-year-old man, ex-smoker, presented to hospital with progressive dyspnea and cough productive of copious amounts of clear sputum. Several months ago, he was treated with antibiotics for presumed pneumonia, yet without significant improvement. Examination revealed oxygen saturation of 90% with bilateral chest crackles. Radiological investigations showed a mixture of multilobar consolidation, peripheral ground glass opacities with septal thickening, and multiple pulmonary nodules (Figure 1).

**Figure 2:**
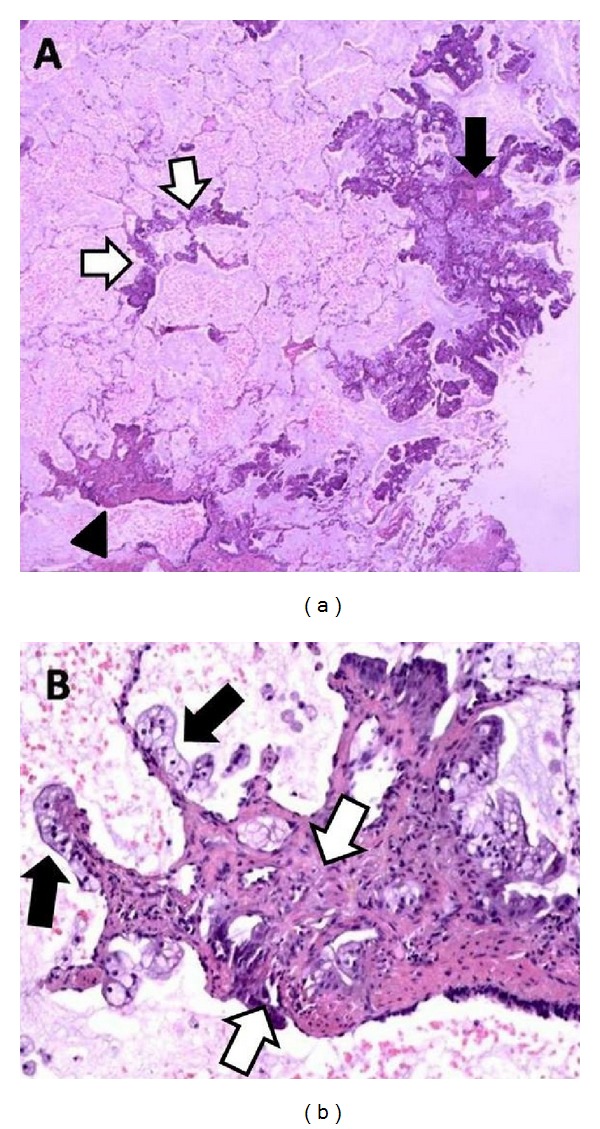
(a) Mucinous adenocarcinoma with lepidic growth pattern (white arrows). Two foci of invasion are seen close to a vessel (black arrow) and around a bronchiole (arrow head). Mucin is present in the alveoli (H&E ×20). (b) The photograph depicts lepidic pattern (black arrows). Invasion of the peribronchiolar tissue is also evident at the center (between white arrows) (H&E ×100).

Bronchoalveolar lavage and transbronchial biopsies suggested malignancy and surgical lung biopsy confirmed the diagnosis of invasive multifocal mucinous adenocarcinoma (Figure 2).

The patient opted for comfort care and died few months later.

## 2. Learning Points

(i) Invasive mucinous adenocarcinoma, formerly known as mucinous bronchioloalveolar carcinoma (BAC), is characterized by its peripheral location, mucin overproduction, lepidic growth pattern (i.e., tumor growth along intact alveolar septa), and propensity to spread via airways and lymphatics.

(ii) Bronchorrhea (sputum production of >100 mL/day) should prompt consideration of invasive mucinous adenocarcinoma in the differential diagnosis. Other causes of bronchorrhea include bronchitis, bronchiectasis, pulmonary contusion, scorpion stings, and organophosphate poisoning.

(iii) The spectrum of radiological findings is broad and includes consolidations, air bronchograms, multifocal solid or subsolid nodules or masses, cavitations, and persistent ground glass opacities. Lower lobe predominance is common [1].

(iv) Treatment options are similar to those for non-small-cell adenocarcinoma with poor prognosis in patients with far-advanced multicentric invasive disease.

(v) Bronchorrhea is usually difficult to treat and can cause hypoxemia secondary to mucus related ventilation/perfusion mismatch, intrapulmonary shunt, and/or diffusion limitation. Successful use of tyrosine kinase inhibitors in those with epidermal growth factor receptor (EGFR) mutation (tested negative in our case), corticosteroids, macrolides, octreotide, and inhaled indomethacin has been reported.

(vi) BAC, a term that should be avoided, has now been replaced by five histologic categories [2]: adenocarcinoma in situ (AIS); minimally invasive adenocarcinoma (MIA); lepidic predominant adenocarcinoma, adenocarcinoma predominantly invasive with some nonmucinous lepidic component; and invasive mucinous adenocarcinoma (formerly mucinous BAC).

(vii) Mucinous adenocarcinoma frequently expresses cytokeratins (CK) 7 and 20 but not thyroid transcription factor-1 (TTF-1), napsin-A, or CDX2. The contradictory expression of CK7 and CDX2 by mucinous subtype and pulmonary metastasis of colorectal carcinoma are important discriminatory factors in their differential diagnosis. Furthermore, mucinous adenocarcinoma is associated with frequent overexpression of K-ras but usually lacks (EGFR) mutations [3].
